# Resilience and yoga training have additive benefits for mental health of intensive care unit nurses

**DOI:** 10.4102/sajpsychiatry.v31i0.2491

**Published:** 2025-08-14

**Authors:** Korhan Kavuran, Ramazan Erdoğan, Amir Dana

**Affiliations:** 1 Department of Physical Education and Sports School, Bitlis Eren University, Bitlis, Turkey; 2 Department of Sport Sciences, Tabriz Branch, Islamic Azad University, Tabriz, Iran

**Keywords:** nurse, ICU, resilience, yoga, mental health

## Abstract

**Background:**

The rigorous demands associated with caring for critically ill patients place intensive care unit (ICU) nurses at a heightened risk for mental health issues.

**Aim:**

This study investigated the individual and combined benefits of two distinct intervention strategies, resilience training and yoga training, on enhancing the mental health of ICU nurses.

**Setting:**

Hospitals located in Bitlis Province, Turkey.

**Methods:**

A sample of 84 participants was chosen and randomly and equally allocated to resilience, yoga, resilience+yoga or the control group. Resilience or yoga training comprised eight sessions, each lasting 45 min. Participants in the combined group completed both resilience and yoga training. Data analysis was conducted using an analysis of variance test.

**Results:**

Engagement in either resilience or yoga training alone led to a significant decrease in depression, anxiety and stress levels (*p* < 0.05). Finally, the resilience + yoga training had additive benefits and resulted in a significantly greater reduction in depression, anxiety and stress levels compared to the individual resilience or yoga training (*p* < 0.05).

**Conclusion:**

Intensive care unit nurses should engage in both resilience and yoga training to reap the associated physical and psychological benefits.

**Contribution:**

This study sheds light on the value of the resilience and yoga combination for the mental health of ICU nurses.

## Introduction

Intensive care units (ICUs) are vital to modern healthcare, providing specialised care for patients with life-threatening conditions.^1,2^ However, the psychological well-being of ICU nurses is a growing concern, with significant rates of anxiety and depression reported globally. In Australia, these issues affect 41.2% and 32.4% of nurses.^3^ A study conducted in Geneva found that 46% of ICU nurses show symptoms of these mental health issues,^4^ and in Saudi Arabia, prevalence rates range from 36.5% to 78.1%.^5^ A recent meta-analysis indicated that 23.2% of ICU nurses suffer from anxiety and 22.8% from depression,^6^ while in Turkey, the rates are 26.2% and 39.8%, respectively.^7^ The impact of these mental health challenges can lead to severe consequences, including burnout, physical health issues, decreased job satisfaction, increased absenteeism and detrimental effects on patient care.^8,9^ Hence, it is crucial to identify the factors that contribute to the alleviation of these disorders and, in turn, enhance the mental health of nursing staff.

Research indicates that various psychological interventions, including relaxation techniques, music therapy and massage, effectively alleviate stress and anxiety.^10,11,12^ There is also a rising interest in cognitive behavioural interventions that take a preventive approach within occupational health. Among the various coping strategies, the cultivation of resilience skills is particularly noteworthy for managing stress effectively.^13,14,15^ The concept of resilience has gained traction among nursing professionals, who define it as the ability to transform adversity into opportunities for personal development and growth.^16,17,18^ Resilience among nurses is recognised as a vital skill that empowers them to navigate and overcome challenges in their work environment. This ability involves adapting to, balancing and managing adverse conditions while seeking effective solutions to various obstacles. By fostering resilience, nurses can mitigate the detrimental impacts of stress, such as burnout, thereby enhancing their overall well-being and supporting both their physical and mental health. Research has indicated a significant inverse relationship between resilience and stress levels among nurses.^19,20^ Hence, nurses may utilise resilience to effectively manage various psychological pressures and environmental stressors. Thus, the primary objective of this study was to examine the impact of resilience training on the mental health of ICU nurses, specifically focusing on its effects in reducing stress, depression and anxiety.

In addition, yoga has been extensively researched and highlighted by scientists as an effective method for achieving mental relaxation and managing stress through both internal and external mechanisms.^21^ This practice serves as a dual-purpose approach, functioning as both a preventive measure and a means of recovery. As one of the oldest known systems of physical and mental discipline, yoga offers a variety of exercises designed to help individuals effectively navigate and mitigate stress. Furthermore, the practice of yoga not only promotes physical fitness but also facilitates emotional equilibrium, boosts self-esteem, cultivates a positive body and social image and encourages relaxation while reinforcing affirmative thoughts.^22,23,24^ Research has demonstrated that yoga can reduce anxiety and improve overall well-being and psychological characteristics such as coherence, resilience and cognitive flexibility,^25,26^ particularly among nurses.^27,28^ Consequently, yoga presents itself as a valuable educational approach for mental health, merging physical activity with mindfulness practices. However, there is a notable lack of research examining its effects on the mental health of ICU nurses, prompting this study to evaluate the effectiveness of yoga training in addressing mental health challenges, specifically stress, depression and anxiety.

As mentioned above, this research investigated the impact of two distinct intervention strategies – resilience training and yoga training – on enhancing the mental health of ICU nurses. However, an intriguing question arises: Does the combination of these approaches result in a ‘doubling’ of the benefits for mental health, or do they merely represent distinct methods of influencing a shared mental health mediator without providing any supplementary value to each other’s impact? Research has investigated the effects of combined interventions that integrate physical exercise with psychotherapeutic methods, particularly resilience training based on cognitive-behavioural therapy. A review by Remskar et al.^29^ indicated that these combined approaches yielded better clinical outcomes for mental health and well-being compared to physical exercise interventions alone. Additionally, a meta-analysis focusing on adults with chronic illnesses revealed that interventions merging physical exercise with cognitive-behavioural therapy led to moderate improvements in depression, anxiety and fatigue.^30^ However, the evidence for the additive benefits of using resilience and yoga together, as opposed to employing either one independently, remains limited. Hence, recognising the potential advantages of integrating resilience and yoga training methods, the study also explored the effects of a combined approach of resilience and yoga training on the mental health of ICU nurses.

## Research methods and design

### Setting

This study was performed in Bitlis Province, Turkey, in the year 2024.

### Design

This study employed a quasi-experimental design characterised by a pretest, post-test and follow-up design alongside a control group. The investigation focused on assessing the impact of two independent variables – resilience training and yoga training – individually or in combination, on the dependent variable of mental health. The implementation process comprised several key steps: (1) random assignment of participants, (2) execution of a pre-test to gather initial data, (3) application of the independent variables to the experimental groups, (4) performance of a post-test to collect subsequent data and (5) execution of a follow-up test. This investigation was carried out under the principles outlined in the Declaration of Helsinki and received approval from the University Ethics Committee (Code: IR.IAU.TNB.REC.1401.059). Ethical protocols were strictly followed, including the acquisition of necessary permissions, obtaining informed consent from the participating nurses and conveying the study’s aims and methodologies. Participants were assured of the confidentiality of the research findings before the commencement of data collection.

### Participants and sampling

The study population comprised all nurses working in the ICU of hospitals located in Bitlis Province, Turkey, in the year 2024. In this research, the study sample was recruited with the collaboration of the hospital director and nursing matron, following the university’s approval for the study. A sample of 84 participants was chosen using a purposive sampling method and randomly allocated to either the experimental groups (Resilience, Yoga, Combined) or the control group, with each group consisting of 21 participants. A power analysis performed with G*Power revealed that a minimum sample size of 15 participants per group was required, based on an alpha level of 0.05 and a power of 0.80. All nurses involved in the study completed it without any withdrawals. The inclusion criteria for participation in the study required that individuals: (1) be employed in an ICU, (2) exhibit a willingness to participate, (3) have no documented health concerns, (4) give informed consent to take part in the research, (5) not have previously engaged in stress management or resilience training programmes, (6) possess a minimum of 3 years of professional experience and (7) hold at least a bachelor’s degree. Conversely, individuals who did not complete the intervention protocol or the questionnaire, along with those who opted out of continuing their participation in the research, were excluded from the study.

### Measure

#### Mental health

The Depression, Anxiety and Stress Scale-21 (DASS-21), created by Lovibond in 1995,^31^ serves as a tool for assessing levels of depression, anxiety and stress. This instrument is structured into three subscales, each containing seven items dedicated to measuring depression, anxiety and stress. The overall score for each subscale is calculated by aggregating the scores of the relevant items. Respondents evaluate each item on a scale ranging from zero (not applicable to me at all) to three (extremely applicable to me). Specifically, items 21, 17, 16, 13, 10, 5 and 3 are associated with depression; items 20, 19, 15, 9, 7, 4 and 2 pertain to anxiety and items 18, 14, 12, 11, 8 and 6 are linked to stress.^23^ Once multiplied by 2, the scoring for each subscale can vary from zero to 42, with higher scores indicating greater levels of distress experienced by the individual. In the context of this research, the Cronbach’s alpha coefficients for the depression, anxiety and stress subscales were found to be 0.92, 0.90 and 0.93, respectively.

### Data collection

Following collaboration with the hospitals, separate meetings were held for each participant group, during which the researcher clarified the study’s objectives and protocols. On the same day, participants signed consent forms to confirm their willingness to take part in the study and completed demographic and mental health questionnaires as a pretest. Those in the experimental groups received detailed information about the training sessions and the specific content relevant to their groups. The intervention commenced 1 week after this introductory meeting. Throughout the study, participants could communicate with the researcher via telephone, email and social media. One week after the training concluded, all participants completed the mental health questionnaire again as a post-test. A follow-up assessment was conducted 1 month later, during which participants filled out the DASS-21 once more. It is noteworthy that the control group did not participate in any training. Finally, no adverse effects were reported in any of the groups involved in the study.

### Intervention

#### Resilience training

Resilience training comprised eight sessions, each lasting 45 min, and was conducted by a professional psychologist. It comprised workshops, question-and-answer sessions and hands-on activities, which were conducted over two separate sessions to enhance attendance among shift nurses. Various educational resources such as films, slides, brochures and handouts were utilised throughout the sessions. Each session maintained consistent methodology and quality, featuring two workshops per week that integrated both theoretical and practical elements focused on developing resilience skills (see Table 1). This material was derived from reputable sources in nursing management and relevant scholarly articles.^13,14^

**TABLE 1:** Content of resilience training.

Session	Content
1	Introduction to the general framework of the topic: An exploration of the concept of resilience An overview of the traits commonly associated with resilient individuals, which include: (1) a sense of happiness, (2) wisdom and insight, (3) a sense of humour, (4) empathy, (5) rational competence, (6) a defined purpose in life and (7) steadfastness. Additionally, a framework for identifying challenging life circumstances and enhancing adaptability and tolerance within the personal domain will be presented.
2	Objective: To gain an understanding of internal supportive elements. The notion of optimism The locus of control To develop an awareness of stress and strategies for managing it Solution: To identify individual strengths and passions, highlight them and be prepared to apply them effectively.
3	Objective: Understanding external support mechanisms. Network of social support Individual accountability and embracing significant roles Cognitive reframing and the development of positive thought processes (recognising the influence of beliefs and thoughts on behaviour and emotions, as well as awareness of cognitive distortions) Solution: A sense of belonging and worth, coupled with an eagerness to engage.
4	Objective: To explore strategies for fostering resilience. Develop and sustain connections with others Highlight the significance of nurturing positive relationships and adopting constructive attitudes towards individuals Embrace change as a fundamental aspect of life Investigate the variations in individual perception, underscoring the critical influence of thoughts and self-talk.
5	Objective: Explore strategies for enhancing resilience. Cultivating a sense of purpose and optimism regarding future possibilities Engaging in proactive measures Understanding cognitive patterns and highlighting the significance of positive thinking in fostering resilience.
6	Objective: To explore ongoing strategies for enhancing resilience. cultivating self-awareness boosting self-esteem promoting self-confidence
7	Objective: Explore additional strategies for fostering resilience. emphasising self-care practices recontextualising stress.
8	Objective: Exploring strategies for enhancing resilience Investigating the concept of meaning and the pursuit of significance Presenting the principles of meaning therapy Highlighting the necessity of attributing meaning to immutable challenges Summary and conclusion

*Sources:* Tamrakar P, Pant SB, Acharya SP. Anxiety and depression among nurses in COVID and non-COVID intensive care units. Nurs Crit Care. 2023;28(2):272–280. https://doi.org/10.1111/nicc.12685; and Bjørnøy I, Rustøen T, Mesina RJS, Hofsø K. Anxiety and depression in intensive care patients six months after admission to an intensive care unit: A cohort study. Intensive Crit Care Nurs. 2023;78:103473. https://doi.org/10.1016/j.iccn.2023.103473

#### Yoga training

Yoga sessions were conducted over eight sessions, each lasting 45 min, led by a qualified yoga instructor. It was conducted over two separate sessions to enhance attendance among shift nurses. The programme commenced with basic exercises, progressively advancing in complexity as participants became proficient in the movements. The instructor initially focused on teaching correct posture, breath regulation and proper alignment. Throughout the sessions, participants engaged in a variety of activities, including deep breathing techniques, upper body stretches, balance exercises, physical postures (Asanas), breathing practices (Pranayama), relaxation techniques (Shavasana) and meditation. Each week, new and more challenging exercises were introduced alongside the previous week’s routines, culminating in a comprehensive practice by the final week. Additionally, the programme incorporated a review of exercises from earlier weeks to reinforce learning and skill development.

#### Combined training

Participants in the combined group, which engaged in both resilience training and yoga, completed a total of eight sessions. Each session was structured to last 90 min, comprising 45 min dedicated to resilience training followed by 45 min of yoga. The resilience and yoga exercises conducted were identical to those performed in the individual groups.

#### Control group

The control group did not receive any intervention initially. However, upon the conclusion of the study, they were provided with an educational package that included materials focused on resilience and yoga.

### Statistical analysis

Data analysis was performed using IBM SPSS Statistics version 27. Descriptive statistics, including frequency, percentage, mean and standard deviation (s.d.), were employed to analyse the distribution of socio-demographic variables within the study sample. In addition, inferential statistics such as the chi-square test and independent t test were employed to analyse gender differences in demographic data. In addition, to compare the pretest scores across different groups, analysis of variance (ANOVA) was utilised. Furthermore, a 4 (GROUP: Resilience, Yoga, Combined and Control) × 3 (TIME: Pretest, Post-test and Follow-up) ANOVA with repeated measures on the last factor was applied to assess the differences between pretest, post-test and follow-up scores among the groups. Finally, Least Significant Difference (LSD) was used as a post-hoc test. A significance level of 0.05 was set for the analyses.

### Ethical considerations

This investigation was carried out under the principles outlined in the Declaration of Helsinki. Ethical clearance to conduct this study was obtained from the Islamic Azad University Research Ethics Committee (No. IR.IAU.TNB.REC.1401.059). Ethical protocols were strictly followed, including the acquisition of necessary permissions, obtaining informed consent from the participating nurses and conveying the study’s aims and methodologies. Participants were assured of the confidentiality of the research findings before the commencement of data collection.

## Results

### Demographic characteristics

Table 2 presents the demographic characteristics of the study sample. Among the 84 participants (mean age = 32.90 years), there were 64 women and 20 men. The age group with the greatest representation was those aged between 28 and 35 years, while the lowest representation was found in the 40- to 45-year age bracket. Regarding marital status, the findings revealed that 38% of participants were single, 58% were married and the remainder were either divorced or widowed. An analysis of the participants’ work experience as a nurse showed that 30% had between 5 and 10 years of experience, whereas 70% had more than 10 years. The average duration of work experience as an ICU nurse was 3.84 years. Furthermore, it was noted that 50% of the participants had children. Lastly, the average body mass index (BMI) of the participants was recorded at 24.67 kg/m^2^.

**TABLE 2:** Demographic characteristics of the study sample.

Variable	Total (*N* = 84)			Men (*N* = 20)			Women (*N* = 64)			Comparison (*p*-value)
	Mean ± s.d.	*n*	%	Mean ± s.d.	*n*	%	Mean ± s.d.	*n*	%	
Age (years)	32.90 ± 3.23	-	-	34.63 ± 4.74	-	-	30.52 ± 3.02	-	-	< 0.001
**Marital status**										
Single	-	22	26	-	6	7	-	16	19	< 0.001
Married	-	58	69	-	13	15	-	45	54	< 0.001
Divorced/widow	-	4	5	-	1	1	-	3	4	< 0.001
**Experience as a nurse (years)**										
5–10	-	2	30	-	6	7	-	19	23	< 0.001
> 10	-	59	70	-	14	16	-	45	54	< 0.001
Experience as an ICU nurse (years)	3.84 ± 0.35	-	-	3.90 ± 0.40	-	-	3.82 ± 0.32	-	-	0.128
**Father/mother**										
Yes	-	42	50	-	11	13	-	31	37	< 0.001
No	-	42	50	-	9	11	-	33	39	< 0.001
BMI kg/m^2^	24.67 ± 1.28	-	-	24.12 ± 1.07	-	-	25.88 ± 1.43	-	-	< 0.001

ICU, intensive care unit; BMI, body mass index; s.d., standard deviation.

### Baseline mental health

Table 3 presents the mean and SD for depression, anxiety and stress levels among nurses categorised by groups. The findings indicate that the mean depression score was 21.35, reflecting a severe degree of depressive symptoms. A significant majority of nurses, specifically 73.8%, reported experiencing moderate to severe depressive symptoms. This pattern was consistently observed across all four groups. Furthermore, the average anxiety score recorded was 16.95, which also points to a severe level of anxiety. In this case, 65.3% of nurses reported moderate to severe anxiety symptoms, with a similar trend evident across the four groups. Lastly, the stress data indicated an average score of 24.47, suggesting a moderate level of stress. Here, 52.5% of nurses reported moderate stress symptoms, again showing comparable results across the groups. Overall, the study’s findings revealed no significant differences among the groups in the pretest, indicating that the mental health status was largely similar across all groups (*p* > 0.05).

**TABLE 3:** Baseline mental health.

Variable	Total (*N* = 84)			Resilience (*N* = 21)			Yoga (*N* = 21)			Resilience+Yoga (*N* = 21)			Control (*N* = 21)			Comparison	
	Mean ± s.d.	*n*	%	Mean ± s.d.	*n*	%	Mean ± s.d.	*n*	%	Mean ± s.d.	*n*	%	Mean ± s.d.	*n*	%	*F*	*p*-value
**Depression**	21.35 ± 5.75	-	-	21.23 ± 5.83	-	-	21.33 ± 6.72	-	-	21.42 ± 5.51	-	-	21.42 ± 5.27	-	-	0.005	0.999
Normal	-	-	-	-	-	-	-	-	-	-	-	-	-	-	-	-	-
Mild	-	7	8.3	-	2	9.5	-	2	9.5	-	1	4.7	-	2	9.5	-	-
Moderate	-	31	36.9	-	7	33.3	-	7	33.3	-	9	42.9	-	8	38.1	-	-
Severe	-	31	36.9	-	9	42.9	-	7	33.3	-	8	38.1	-	7	33.3	-	-
Extremely severe	-	15	17.9	-	3	14.3	-	5	23.8	-	3	14.3	-	4	19.1	-	-
**Anxiety**	16.95 ± 4.59	-	-	16.57 ± 5.06	-	-	17.19 ± 4.55	-	-	17.00 ± 4.54	-	-	17.04 ± 4.48	-	-	0.068	0.977
Normal	-	-	-	-	-	-	-	-	-	-	-	-	-	-	-	-	-
Mild	-	3	3.5	-	2	9.5	-	-	-	-	1	4.7	-	-	-	-	-
Moderate	-	19	22.6	-	4	19.1	-	5	23.8	-	5	23.8	-	5	23.8	-	-
Severe	-	36	42.7	-	9	42.9	-	9	42.9	-	8	38.1	-	10	47.7	-	-
Extremely severe	-	26	30.2	-	6	28.5	-	7	33.3	-	7	33.3	-	6	28.5	-	-
**Stress**	24.47 ± 6.58	-	-	24.61 ± 7.92	-	-	24.90 ± 6.34	-	-	24.00 ± 6.27	-	-	24.38 ± 6.12	-	-	0.068	0.977
Normal	-	-	-	-	-	-	-	-	-	-	-	-	-	-	-	-	-
Mild	-	14	16.7	-	6	28.5	-	3	14.3	-	3	14.3	-	2	9.5	-	-
Moderate	-	44	52.5	-	8	38.1	-	11	52.4	-	12	57.1	-	13	61.9	-	-
Severe	-	15	17.6	-	2	9.5	-	4	19.0	-	5	23.8	-	4	19.0	-	-
Extremely severe	-	11	13.2	-	5	23.8	-	3	14.3	-	1	4.7	-	2	9.5	-	-

### Effects of different interventions on mental health

#### Depression

The data presented in Table 4 summarise the outcomes of the comparative analysis of depression scores recorded during the study stages across various groups. The results reveal a statistically significant impact attributed to GROUP (*F* = 10.527, *p* < 0.001). Here, the results of multiple comparisons indicate that the combined resilience and yoga training led to a significantly greater enhancement in depression scores when contrasted with the separate resilience or yoga training interventions (*p* < 0.05). Additionally, participation in either resilience or yoga training alone was linked to a significant reduction in depression levels (*p* < 0.05). Notably, no significant differences were observed between the individual resilience and yoga training groups (*p* = 0.974).

**TABLE 4:** Results of repeated measures analysis of variance for depression.

Source	Type III sum of square	*df*	Mean square	*F*	Sig.	Partial Eta squared
Group	2055.536	3	685.179	10.527	< 0.001	0.283
Time	1841.484	2	920.742	264.072	< 0.001	0.767
Group*time	1036.643	6	172.774	49.552	< 0.001	0.650

*df*, degrees of freedom; Sig., significance.

#### Anxiety

The information contained in Table 5 summarises the results from the comparative analysis of anxiety scores obtained during the study stages across various groups. The results reveal a statistically significant impact for GROUP (*F* = 5.715, *p* < 0.001). Here, the results of multiple comparisons indicate that the combination of resilience and yoga training led to a significantly greater decrease in anxiety scores when compared to those who underwent either resilience training or yoga training alone (*p* < 0.05). In addition, participation in either resilience or yoga training independently was linked to a significant reduction in anxiety levels (*p* < 0.05). Notably, no significant differences were found between the individual resilience training and yoga training groups (*p* = 0.968).

**TABLE 5:** Results of repeated measures analysis of variance for anxiety.

Source	Type III sum of square	*df*	Mean square	*F*	Sig.	Partial Eta squared
Group	752.996	3	250.999	5.715	< 0.001	0.176
Time	599.389	2	299.694	165.240	< 0.001	0.674
Group*time	377.087	6	62.848	34.652	< 0.001	0.656

*df*, degrees of freedom; Sig., significance.

#### Stress

The data contained in Table 6 summarise the findings from the comparative analysis of stress scores collected during the study stages across different groups. The analysis demonstrates a statistically significant effect for GROUP (*F* = 7.056, *p* < 0.001). Here, the results of multiple comparisons indicate that the integration of resilience and yoga training resulted in a significantly greater decrease in stress scores compared to those who participated in either resilience training or yoga training alone (*p* < 0.05). Moreover, participation in either resilience training or yoga practice was linked to a significant decrease in stress levels (*p* < 0.05). Importantly, no significant differences were found between the individual resilience training and yoga training groups (*p* = 0.906).

**TABLE 6:** Results of repeated measures analysis of variance for stress.

Source	Type III sum of square	*df*	Mean square	*F*	Sig.	Partial Eta squared
Group	2003.472	3	667.824	7.056	< 0.001	0.209
Time	1804.151	2	902.075	197.638	< 0.001	0.712
Group*time	918.230	6	153.038	33.530	< 0.001	0.557

*df*, degrees of freedom; Sig., significance.

## Discussion

This study investigated individual and combined benefits of two distinct intervention strategies – resilience training and yoga training – on enhancing the mental health of ICU nurses. The preliminary results reveal that most of the ICU nurses involved in this study exhibited moderate to severe symptoms of depression, anxiety and stress before participating in the interventions. These outcomes are consistent with earlier research and imply that employment as an ICU nurse may lead to significant psychological distress.^5,6,7,8,9^

In terms of individual benefits, the findings of this research indicated that participation in a resilience training programme resulted in enhanced mental health among ICU nurses, with these benefits persisting for a minimum of 1 month following the completion of the training. The findings align with recent studies^13,14,15,16^ and suggest that resilience training has a beneficial effect on the mental health of ICU nurses. To interpret these findings, it can be stated that resilience training can be understood as a mechanism that fosters a belief in one’s capabilities and cultivates an optimistic outlook on life.^14^ It serves as a foundational element that assists individuals in bridging the gap between their inherent strengths and their limitations.^13^ Through supportive encouragement, individuals gain insight into their core values and recognise their strengths and resources. Consequently, individuals exhibiting high resilience tend to demonstrate greater behavioural flexibility and establish more effective interpersonal relationships.^15^ Furthermore, resilience training equips individuals with the tools to cultivate resilience within themselves, thereby protecting their mental health and overall well-being from stress and other detrimental influences.^14,32,33^ Accordingly, engagement in resilience group training sessions facilitated ICU nurses in acknowledging their challenges, such as anxiety, depression and stress and rationally addressing them. In addition, research findings indicate that resilience training may lead to a decrease in cortisol levels, a biomarker associated with stress responses.^34^ Thus, resilience training emerges as a vital strategy within positive psychology, particularly for ICU nurses, as it can significantly enhance mental health by bolstering internal resistance and mitigating the adverse effects of depression, anxiety and stress while potentially preventing future physical and psychological disorders.

Furthermore, the findings of this research indicated that participation in a yoga training programme resulted in enhanced mental health among ICU nurses, with these benefits persisting for a minimum of 1 month following the completion of the training. The findings align with recent studies^24,25,26,27^ and suggest that yoga training has a beneficial effect on the mental health of ICU nurses. To interpret these findings, it can be stated that yoga has been demonstrated to effectively alleviate pain, illness and chronic disabilities.^25,28^ Engaging in yoga training induces a range of physiological changes within the body that mitigate mental stress.^35,36^ The practice of conscious breathing is particularly beneficial in diminishing anxiety and stress levels. The beneficial impact of yoga on anxiety and stress management can also be understood through the lens of attentional bias techniques.^37^ Furthermore, research indicates that yoga can lower cortisol levels and decrease perceived stress among individuals,^38,39^ potentially explaining the observed reduction in anxiety and stress among ICU nurses. This theory posits that an imbalance in neurotransmitters, specifically dopamine – associated with reward and pleasure – and serotonin, which is linked to feelings of happiness, contributes to the onset of depression. Recent studies utilising positron emission tomography have demonstrated that engaging in yoga can elevate dopamine levels in the striatum.^40^ Additionally, depression is characterised by reduced levels of serum brain-derived neurotrophic factor (BDNF) and elevated cortisol levels. Engaging in yoga has been shown to enhance serum BDNF levels while simultaneously reducing cortisol levels, thereby exhibiting antidepressant properties.^41^ This characteristic of yoga minimises resistance to treatment for psychological distress among these individuals.^26^ Consequently, engaging in yoga may help to dismantle certain barriers to mental health care. Additionally, participation in yoga can foster positive experiences for ICU nurses, enhancing their social interactions. The endeavour to learn and practice a new skill also cultivates a sense of mastery, which can bolster self-efficacy and positively influence the mood of ICU nurses.

Finally, the research findings suggest that engaging in a combined training programme that incorporates both resilience and yoga significantly enhances mental health outcomes for ICU nurses, surpassing the individual effects of each training modality. Notably, these improvements were observed to last for at least 1 month after the training concluded. The results indicated that while the mental health benefits of resilience and yoga training were comparable when considered separately, their integration produced additive effects. In both mental health assessments, including the post-test and follow-up evaluations, the group participating in the combined resilience and yoga training demonstrated superior performance compared to all other groups. The ‘double advantage’ of exposure to the resilience and yoga training programme effect suggests that the mechanisms underlying each variable may be at least partially independent. This study represents, to the best of our understanding, the inaugural investigation into the synergistic impact of resilience training and yoga on the mental health of ICU nurses. Consequently, it is not feasible to draw comparisons between the findings of this research and those of prior studies. Additionally, the specific mechanisms underlying the observed additive effect of the combined interventions on the mental health of ICU nurses remain ambiguous. Nevertheless, while this research did not delve into the potential mechanisms contributing to the additive effect of resilience training and yoga, it is noteworthy that both interventions, beyond their psychobiological benefits, share a common influence in lowering cortisol levels – an established marker of stress responses – which may facilitate improvements in mental health,^34,38,41^ including reductions in depression and anxiety. Thus, by highlighting the additive benefits of engaging in both resilience and yoga training for the mental health of ICU nurses, this study advocates for future inquiries to explore the underlying mechanisms of this additive effect, particularly in cortisol level reduction.

The current study has several limitations that should be acknowledged. Notably, we did not adjust for baseline demographic differences in our statistical analysis, which suggests that future research should incorporate these adjustments when comparing groups. Additionally, the absence of blinding in this study may introduce bias, highlighting the need for blinding in subsequent investigations to enhance the reliability of results. Furthermore, the relatively small sample size limits the generalisability of our findings, indicating that larger sample sizes should be employed in future studies to improve external validity. Lastly, the reliance on self-reported data raises concerns about potential biases, such as social desirability and recall bias, which future research should aim to mitigate.

## Conclusion

This study is, to our knowledge, the most extensive systematic investigation into the effects of resilience and yoga on mental health within a high-risk occupational group. Our results demonstrate that a combined intervention of resilience and yoga significantly alleviates symptoms of depression, anxiety and stress among nurses working in the ICU. Furthermore, the findings underscore the importance of integrating resilience and yoga practices into daily routines for enhanced mental well-being of ICU nurses. A deeper understanding of the mental health of professionals working in high-stress settings, such as ICU nurses, will be enriched by ongoing research utilising both behavioural and biological approaches to assess the relative efficacy of, and the underlying processes associated with, other significant variables. Practically, based on the findings of this study, ICU nurses should engage in both resilience training and yoga to reap the associated mental health benefits.

## References

[1] Yarahmadi S, Soleimani M, Gholami M, Fakhr-Movahedi A, Madani SMS. Health disparities in service delivery in the intensive care unit: A critical ethnographic study. Nurs Crit Care. 2024;30(3):e13170. 10.1111/nicc.13170 39385472

[2] Wang L, Chen Y, Yu H, et al. The experiences of newly qualified nurses in intensive care unit: A qualitative meta-synthesis. Front Med (Lausanne). 2024;11:1458845. 10.3389/fmed.2024.1458845 39540044 PMC11557346

[3] Zakeri H, Mahtosh P, Radmehr M, et al. Pain management strategies in intensive care unit: Challenges and best practice. Galen Med J. 2024;13:1–9. 10.31661/gmj.v13i.3264 PMC11368475 39224543

[4] Ünver S, Yildirim M, Akbal S, Sever S. Challenges experienced by cardiac intensive care nurses during first out-of-bed patient mobilization after open-heart surgery: A descriptive phenomenological qualitative study. J Adv Nurs. 2024;80(11):4616–4628. 10.1111/jan.16091 38318643

[5] Teng M, Yuan Z, He H, Wang J. Levels and influencing factors of mental workload among intensive care unit nurses: A systematic review and meta-analysis. Int J Nurs Pract. 2024;30(4):e13167. 10.1111/ijn.13167 37259643

[6] Zeng LN, Cai H, Gao F, et al. Assessment of mental health status among Chinese nursing staff in the intensive care unit: A network analysis. J Res Nurs. 2023;28(4):285–298. 10.1177/17449871231172408 37534263 PMC10392721

[7] Hall CE, Milward J, Spoiala C, et al. The mental health of staff working on intensive care units over the COVID-19 winter surge of 2020 in England: A cross sectional survey. Br J Anaesth. 2022;128(6):971–979. 10.1016/j.bja.2022.03.016 35465953 PMC8942706

[8] Heesakkers H, Zegers M, Van Mol MMC, Van Den Boogaard M. Mental well-being of intensive care unit nurses after the second surge of the COVID-19 pandemic: A cross-sectional and longitudinal study. Intensive Crit Care Nurs. 2023;74:103313. 10.1016/j.iccn.2022.103313 36153185 PMC9393155

[9] Greenberg N, Weston D, Hall C, Caulfield T, Williamson V, Fong K. Mental health of staff working in intensive care during COVID-19. Occup Med (Lond). 2021;71(2):62–67. 10.1093/occmed/kqaa220 33434920 PMC7928568

[10] Saravanabavan L, Sivakumar MN, Hisham M. Stress and burnout among intensive care unit healthcare professionals in an Indian Tertiary Care Hospital. Indian J Crit Care Med. 2019;23(10):462–466. 10.5005/jp-journals-10071-23265 31749555 PMC6842838

[11] Vahedian-Azimi A, Hajiesmaeili M, Kangasniemi M, et al. Effects of stress on critical care nurses: A National Cross-Sectional study. J Intensive Care Med. 2019;34(4):311–322. 10.1177/0885066617696853 29277137

[12] Huang H, Xia Y, Zeng X, Lü A. Prevalence of depression and depressive symptoms among intensive care nurses: A meta-analysis. Nurs Crit Care. 2022;27(6):739–746. 10.1111/nicc.12734 34989060

[13] Tamrakar P, Pant SB, Acharya SP. Anxiety and depression among nurses in COVID and non-COVID intensive care units. Nurs Crit Care. 2023;28(2):272–280. 10.1111/nicc.12685 34580949 PMC8662271

[14] Bjørnøy I, Rustøen T, Mesina RJS, Hofsø K. Anxiety and depression in intensive care patients six months after admission to an intensive care unit: A cohort study. Intensive Crit Care Nurs. 2023;78:103473. 10.1016/j.iccn.2023.103473 37354695

[15] Özgencil E, Ünal N, Oral M, Okyavuz Ü, Alanoglu Z, Tulunay M. Depression and burnout syndrome in intensive care unit nurses. Crit Care. 2004;8(Suppl 1):P340. 10.1186/cc2807

[16] Zhang Y, Wu C, Ma J, et al. Relationship between depression and burnout among nurses in Intensive Care units at the late stage of COVID-19: A network analysis. BMC Nurs. 2024;23(1):224. 10.1186/s12912-024-01867-3 38561758 PMC10983623

[17] Alzailai N, Barriball L, Xyrichis A. Burnout and job satisfaction among critical care nurses in Saudi Arabia and their contributing factors: A scoping review. Nurs Open. 2021;8(5):2331–2344. 10.1002/nop2.843 33760366 PMC8363385

[18] Myhren H, Ekeberg O, Stokland O. Job satisfaction and burnout among intensive care unit nurses and physicians. Crit Care Res Pract. 2013;2013:786176. 10.1155/2013/786176 24303211 PMC3835606

[19] Zhang M, Murphy B, Cabanilla A, Yidi C. Physical relaxation for occupational stress in healthcare workers: A systematic r eview and network meta-analysis of randomized controlled trials. J Occup Health. 2021;63(1):e12243. 10.1002/1348-9585.12243 34235817 PMC8263904

[20] Cooke M, Holzhauser K, Jones M, Davis C, Finucane J. The effect of aromatherapy massage with music on the stress and anxiety levels of emergency nurses: Comparison between summer and winter. J Clin Nurs. 2007;16(9):1695–1703. 10.1111/j.1365-2702.2007.01709.x 17727588

[21] Finnerty R, Zhang K, Tabuchi RA, Zhang K. The use of music to manage burnout in nurses: A systematic review. Am J Health Promot. 2022;36(8):1386–1398. 10.1177/08901171221105862 35633074 PMC9619252

[22] Zhai X, Ren LN, Liu Y, Liu CJ, Su XG, Feng BE. Resilience training for nurses: A meta-analysis. J Hosp Palliat Nurs. 2021;23(6):544–550. 10.1097/NJH.0000000000000791 34313624

[23] Abbasalizadeh M, Farsi Z, Sajadi SA, Atashi A, Fournier A. The effect of resilience training with mHealth application based on micro-learning method on the stress and anxiety of nurses working in intensive care units: A randomized controlled trial. BMC Med Educ. 2024;24(1):442. 10.1186/s12909-024-05427-w 38658914 PMC11041025

[24] Hasani H, Zarei B, Danaei Z, Mahmoudirad G. Comparing the effect of resilience skills training and metacognitive therapy on job stress in nurses: An experimental study. Iran J Nurs Midwifery Res. 2022;27(5):377–384. 10.4103/ijnmr.ijnmr_59_21 36524134 PMC9745846

[25] Wiig S, Lyng HB, Guise V, et al. From theory to policy in resilient health care: Policy recommendations and lessons learnt from the resilience in health care research program. J Patient Saf. 2024;20(7):e109–e114. 10.1097/PTS.0000000000001258 39115385

[26] Polk LV. Toward a middle-range theory of resilience. ANS Adv Nurs Sci. 1997;19(3):1–13. 10.1097/00012272-199703000-00002 9055026

[27] Alshehry AS. Association of personal and professional factors, resilience and quality of life of registered nurses in a university medical city in the Kingdom of Saudi Arabia. PLoS One. 2024;19(9):e0310263. 10.1371/journal.pone.0310263 39255268 PMC11386420

[28] Suazo Galdames I, Molero Jurado MDM, Fernández Martínez E, Pérez-Fuentes MDC, Gázquez Linares JJ. Resilience, burnout and mental health in nurses: A latent mediation model. J Clin Med. 2024;13(10):2769. 10.3390/jcm13102769 38792311 PMC11121760

[29] Rayani AM, Alodhailah AM, Alreshidi SM. A cross-sectional study of resilience and well-being among nursing students in Saudi Arabia. SAGE Open Med. 2024;12:20503121241245224. 10.1177/20503121241245224 38623476 PMC11017810

[30] Abdulmohdi N. The relationships between nurses’ resilience, burnout, perceived organisational support and social support during the second wave of the COVID-19 pandemic: A quantitative cross-sectional survey. Nurs Open. 2024;11(1):e2036. 10.1002/nop2.2036 38268251 PMC10697858

[31] Alexander GK, Rollins K, Walker D, Wong L, Pennings J. Yoga for self-care and burnout prevention among nurses. Workplace Health Saf. 2015;63(10):462–470; quiz 471. 10.1177/2165079915596102 26419795

[32] Cocchiara RA, Peruzzo M, Mannocci A, et al. The use of yoga to manage stress and burnout in healthcare workers: A systematic review. J Clin Med. 2019;8(3):284. 10.3390/jcm8030284 30813641 PMC6462946

[33] Anderson R, Mammen K, Paul P, Pletch A, Pulia K. Using yoga nidra to improve stress in psychiatric nurses in a pilot study. J Altern Complement Med. 2017;23(6):494–495. 10.1089/acm.2017.0046 28530462

[34] Rostami K, Ghodsbin F. Effect of yoga on the quality of life of nurses working in intensive care units. randomized controlled clinical trial. Invest Educ Enferm. 2019;37(3):e06. 10.17533/udea.iee.v37n3e06 PMC7871494 31830404

[35] Yılmaz R, Çevik Kaya K. The effect of laughter yoga applied to intensive care nurses on their perceived stress, job motivation, and mental well-being: Randomized controlled study. Clin Nurse Spec. 2024;38(5):229–236. 10.1097/NUR.0000000000000839 39159324

[36] Hilcove K, Marceau C, Thekdi P, Larkey L, Brewer MA, Jones K. Holistic nursing in practice: Mindfulness-based yoga as an intervention to manage stress and burnout. J Holist Nurs. 2021;39(1):29–42. 10.1177/08980101221127086 32460584

[37] Sis Çelik A, Yarali S. The effect of laughter yoga on the psychological resilience and sleep quality of nurses during the pandemic: A randomized controlled trial. Altern Ther Health Med. 2023;29(5):146–152. 37023316

[38] Mathad MD, Pradhan B, Sasidharan RK. Effect of yoga on psychological functioning of nursing students: A randomized wait list control trial. J Clin Diagn Res. 2017;11(5):KC01–KC05. 10.7860/JCDR/2017/26517.9833 PMC5483709 28658807

[39] Marijanović I, Kraljević M, Buhovac T, et al. Use of the Depression, Anxiety and Stress Scale (DASS-21) questionnaire to assess levels of depression, anxiety, and stress in healthcare and administrative staff in 5 oncology institutions in Bosnia and Herzegovina during the 2020 COVID-19 pandemic. Med Sci Monit. 2021;27:e930812. 10.12659/MSM.930812 33867520 PMC8063632

[40] Foster K, Cuzzillo C, Furness T. Strengthening mental health nurses’ resilience through a workplace resilience programme: A qualitative inquiry. J Psychiatr Ment Health Nurs. 2018;25(5–6):338–348. 10.1111/jpm.12467 29920873

[41] Grabbe L, Higgins MK, Baird M, Pfeiffer KM. Impact of a resiliency training to support the mental well-being of front-line workers: Brief report of a Quasi-experimental study of the community resiliency model. Med Care. 2021;59(7):616–621. 10.1097/MLR.0000000000001535 33827106 PMC8191373

